# Supplementary material for: Overview of Diagnostic Methods, Disease Prevalence and Transmission of Mpox (Formerly Monkeypox) in Humans and Animal Reservoirs
Source: Microorganisms. 2023 Apr 30;11(5):1186. doi: 10.3390/microorganisms11051186 (PMC10223876; doi:10.3390/microorganisms11051186)
Supplement: Supplementary file 1 [file microorganisms-11-01186-s001.zip › Supplementary Table S1.pdf]

**Supplementary Table S1**: Data on natural Mpox infections in humans, reported in selected articles and case reports, published up to 2 September 2022

| Country | Duration of study | Clinical disease symptoms | Clinical samples used for Mpox or OPXV detection | Techniques used for Mpox or OPXV detection | Summary of results (Number of Mpox or OPXV positive samples detected) | Reported history of contact with animals/ Notes | Reported transmission status | Citations |
|---|---|---|---|---|---|---|---|---|
| DRC | 1970 | Fever, rash | Crust | Virus isolation | Virus isolation (1/1) | None reported | None reported | [57] |
| | 1980-1984 | Skin lesions, rash | Vesicular and pustular fluid or scab, blood | Virus isolation, EM, HI, ELISA IgG, RIA | Serology (44/559; using sera). Virus isolation and EM (48/559; using lesions) | None reported | Human-to-human | [62] |
| | 1982 | Fever, pustular rash, inguinal lympaadenopathy | Crusted scab | Virus isolation, EM | Virus isolation (1/1); EM (1/1) | Bitten by a chimpanzee | Chimpanzee-to-human | [141] |
| | 1983 | Pustular rash, lymphadenopathy, fever | Skin lesions, blood | Virus isolation, RIA | RIA (2/5; using blood). Virus isolation (3/5; using skin lesions) | History of feeding upon red colobus monkeys in a family, where three people were Mpox positive | Human-to-human, hospital acquired | [173] |
| | 1996 | Skin lesions | Crusted scabs, vesicular fluid, blood | PCR, virus isolation, western blot, ELISA IgM | Mpox-PCR (3/4; using crusted scabs); Mpox-IgM ELISA (5/6); OPXV-western blot (10/11); Virus isolation (2/3; using vesicular fluid) | The villagers had a history of hunting squirrels | Human-to-human | [143] |

| | | | | | | | | |
|---|---|---|---|---|---|---|---|---|
| | 1996-1997 | Rash, skin lesions, fever, lymphadenopathy | Skin lesions, blood | Mpox-PCR, virus isolation, HI, neutralization assay, western blot, Sanger sequencing for HA gene | Active cases: Mpox-PCR and virus isolation (7/7; using skin lesions); Sanger sequencing (7/7; Mpox HA gene); VZV-PCR (2/7; using skin lesions); OPXV antibodies in blood: Western blot (6/7); HI (3/7); neutralization assay (2/7); VZV-IgM (5/7). Retrospective cases- OPXV antibodies in blood: western blot (49/72); HI (51/70); neutralization assay (39/72) | Humans feeding upon several wild animals, including squirrels | Squirrel-to-human, human-to-human | [113] |
| | 2001 | Fever, enlarged lymph nodes, lesions | Swabs, crusted scabs | Mpox-PCR, VZV-PCR, EM, virus isolation | Mpox-PCR (7/14); EM (5/14; poxvirus); Virus isolation (4/14; poxvirus) VZV-PCR (5/14); EM (1/14; Herpes virus) | None reported | None reported | [142] |
| | 2001-2004 | Skin lesions, vesicular rash | Crusted scabs, vesicle fluid | OPXV-specific PCR, virus isolation, Sanger sequencing | OPXV-PCR (52/136); Virus isolation (48/136) | None reported | None reported | [115] |

| | | | | | | | | |
|---|---|---|---|---|---|---|---|---|
| | 2005-2007 | Rash | Vesicle fluid, scabs | Illumina Genome Analyzer IIx, Illumina MiSeq, Sanger sequencing | Genome sequencing (23/29) | None reported | Human-to-human | [9] |
| | 2005-2008 | Fever, vesicular-pustular rash | Lesion swab, crust samples | Mpox-real time PCR, VZV-real time PCR | Real-time PCR (633/1286; Mpox-positive only); (152/1286; co-infected with Mpox and VZV); (278/1286; VZV positive only); (95/1286; negative for Mpox and VZV) | No contact with animals | None reported | [88] |
| | 2007-2011 | Skin lesions and rash in patients. Miscarriage of pregnancy, foetal death | Blood, peritoneal fluid of fetus, tissues of the fetus and placenta | PCR, Real-time PCR, IHC | PCR (4/4); Real-time PCR (4/4); IHC (1/1) | None reported | Mother-to-foetus | [40] |
| | 2008-2009 | Skin lesions and rash | Vesicular or pustular exudates on blotting paper, crusted scabs | OPXV-real-time PCR, Mpox-PCR-RFLP, pyrosequencing | OPXV-real time PCR and Mpox-PCR-RFLP (13/25) | None reported | Human-to-human | [170] |
| | 2009-2014 | Rash, lesions, fever, lymphadenopathy, crusts on palms and hands | Lesion swabs, crust samples, blood | Mpox-qPCR, VZV- qPCR, virus isolation, EM | Mpox-PCR and VZV-PCR dual positive (40/427); EM (2/4; poxvirus, 2/4; herpesvirus virions) | No contact with animals | None reported | [8] |
| | 2009-2014 | Fever, rash, nausea, cough, chills, sore throat, mouth ulcers, | Vesicular swabs, crust | OPXV-real-time PCR, Mpox-real-time PCR, | Mpox-real-time PCR (333/752); | None reported | Human-to-human | [87] |

| | | | | | | | | |
|---|---|---|---|---|---|---|---|---|
| | | headache, lymphadenopathy, pruritis, malaise, fatigue, sensitivity to light, conjunctivitis, bedridden | | VZV-real-time PCR | VZV-real-time PCR (383/752) | | | |
| | 2010 | Vesicular pustular eruptions with lesions, fever, lymphadenopathy (inguinal, axillary, and/or cervical), and/or pustules or crusts on the palms of the hands or soles of the feet | Vesicular or pustular lesion fluid, lesion crust, vesicular or pustular lesion roof | MPX/OPX gene expert assay (multiplex real-time PCR), Mpox-specific real time-PCR | Mpox-real time PCR (25/50; crust specimens); Mpox-real time PCR (63/114; vesicular fluid specimens). MPX/OPX gene Xpert assay: (23/23; crust specimens); (60/61; vesicular swabs) | None reported | None reported | [128] |
| | 2011-2014 | Fever, rash, cough, swollen face | Vesicular swab, crust, blood | PCR, Illumina sequencing | Mpox-PCR (3/6); VZV-PCR (1/6); Illumina sequencing-partial Mpox genes (3/3) | History of feeding upon monkeys | Animal-to-human, via bush meat | [114] |
| | 2011-2015 | Fever, coughing, lymphadenopathy, dysphagia, rash, headache, skin lesions, buccal ulcers, conjunctivitis, photophobia | Vesicular swab exudates, crust, blood | OPXV- real-time PCR, Mpox- real-time PCR, VZV- real-time PCR | OPXV- real-time PCR (1057/1658); Mpox- real-time PCR (775/1057); Mpox- real-time PCR and VZV-real-time PCR (169/1057) | Contact with animals due to occupational exposure or food habits | Animal-to-human; human-to-human | [85] |
| | 2013 | Fever, vesicular rash | Vesicular or ocular swab, crust specimens | OPXV- real-time PCR | OPXV- real-time PCR (50/63) | None reported | Human-to-human | [171] |

| | | | | | | | | |
|---|---|---|---|---|---|---|---|---|
| | 2013 | Fever, vesicular rash | Vesicular or ocular swab, crust specimens | OPXV- real-time PCR | OPXV- real-time PCR (20/63) | None reported | Human-to-human | [197] |
| Nigeria, Liberia, Sierra Leone | 1970-1971 | vesiculopustular rash, lesions, fever, sore throat, malaise, headache, severe prostration, sweating | Blood, crust | Virus isolation, EM, agar gel test, HI | HI (2/6; Vaccinia virus); EM (3/3; poxvirus particles in vesicular fluid samples); Virus isolation (3/3; vesicular fluid, and 3/4; crust specimens); agar-gel test (3/4; crust specimens) | Patients either ate monkeys or had contact with internal organs after butchering | Monkey-to-human, Human-to-human | [61] |
| Nigeria, Liberia, Sierra Leone | 1970-1971 | Skin lesions, crusts | Vesicular fluid, crust | Virus isolation, EM, agar gel test | Virus isolation (1/1; skin lesions, 4/4; vesicular fluid, 3/5; crust specimens. EM (1/1; skin lesions, 4/4; vesicular fluid, 3/3; crust. Agar gel test (1/1; skin, 1/1; vesicular fluid, 3/3; crust. | None reported | None reported | [59] |
| Nigeria, DRC, Liberia, Ivory Coast, Sierra Leone | 1970-1979 | Fever, rash, skin lesions | Skin scrapings, blood | Virus isolation, EM, precipitation-in-gel test, HI, complement fixation, RIA | Virus isolation (32/47); EM (38/47); Precipitation in-gel test (20/47); RIA (17/47); Complement fixation (3/47) | One child who developed a rash had contact with a dead squirrel two weeks before onset of disease symptoms | Human-to-human | [60] |
| Nigeria, Ivory Coast | 1976 | None reported | Blood | OPXV-IFA, Mpox-IFA, virus isolation | OPXV-IFA (3/3); Mpox-IFA (3/3); | Contact with monkeys | Monkey-to-human | [126] |

| | | | | | | | | |
|---|---|---|---|---|---|---|---|---|
| | | | | | virus isolation (1/3) | | | |
| Nigeria | 2017 | Fever, rash, headache, malaise, sore throat | Blood, lesion swab, crust, pus, blood on skin | Real-time PCR, ELISA IgM, genomic sequencing | Mpox- confirmed (42/107). No details on positivity rate using different methods. | Contact with monkeys | Human-to-human, within family | [83] |
| | 2017-2018 | Vesiculopustular rash, fever, headache, pruritus, lymphadenopathy | Blood, lesion swab, crust (dried rash debris-serum, pus or blood on the skin surface) | Mpox-real time PCR, Mpox-ELISA IgM, OPXV-ELISA IgG, virus isolation, Illumina MiSeq, Sanger sequencing | Mpox-real-time PCR (104/253); Mpox-ELISA IgM (14/253); OPXV-ELISA IgG (15/135); Genome sequencing (7 Mpox whole genomes) | Contact with monkeys, rodents, and wild animals for bush meat | Human-to-human, in households and a prison facility, and to healthcare workers, animal-to-human | [12] |
| Singapore, United Kingdom, Israel | 2018 | Pustular rash | Pustule swab | Virus isolation, Illumina MiSeq, Nanopre MinION sequencing | Virus isolation (6/6); Illumina MiSeq (6/6); Nanopore MinION (6/6) | None reported | Human-to-human (First exportation of Mpox from Africa via air travel) | [68] |
| Singapore | 2019 | Fever, muscle aches, chills, nodular skin lesions | Blister fluid | EM, Mpox-PCR, Sanger sequencing | Mpox- PCR (1/1); EM (1/1; poxvirus psrticles); Sanger sequencing (1/1) | None reported | None reported | [198] |
| | 2019 | Fever, chills, myalgia, rash, vesiculo-pustular lesions, cervical and inguinal lymphadenopathy | Blood, lesion swab, vesicle fluid | OPXV- PCR, Mpox- PCR, EM, Illumina MiSeq | OPXV- PCR (1/1); Mpox-PCR (1/1); EM (1/1; poxvirus particles); Illumina MiSeq (1/1) | Traveller fed upon barbequed bushmeat, suspected reason of transmission | None reported | [70] |

| | | | | | | | | |
|---|---|---|---|---|---|---|---|---|
| Central African Republic | 2001-2018 | Skin rash | Blood, scab, pus | virus isolation, PCR, real-time PCR, Illumina MiSeq | PCR (10/10); Real-time PCR (10/10); virus isolation (10/10); Illumina MiSeq (10/10) | Contact with wild reservoirs | Animal-to-human, Human-to-human | [112] |
| | 2012 | Fever, myalgia, skin lesions, oedema of hands,<br>lower legs and feet, weakness | Vesicular fluid, lesion biopsy, blood | Mpox- real-time PCR | Mpox- real-time PCR (2/2) | Contact with squirrels through hunting. One patient was bitten by a squirrel | Squirrel to human through biting or contact | [6] |
| | 2016 | Fever, skin rash, pruritus and cervical and/or<br>inguinal adenopathy | Blood | PCR | PCR (3/7) | Index case patient fed upon a dead squirrel (*Xerus erythropus*) | Squirrel-to-human, human-to-human | [158] |
| | 2018 | Maculopapular rash and lesions, fever | Blood or pus | PCR, real-time PCR, ELISA IgG | PCR (5/5); Real-time PCR (5/5); ELISA IgG (6/11) | Contact with three small mammals through butchering | Human-to-human. Intrafamilial transmission | [3] |
| Republic of Congo | 2003 | Fever, rash, headache, malaise | Lesion swabs, blood | OPXV-PCR, Mpox-PCR OPXV-ELISA (IgG, IgM) | OPXV-PCR (4/4); Mpox-PCR (4/4); OPXV-ELISA IgM (3/8); OPXV-ELISA IgG (3/8) | One patient had a pet monkey (*Cercopithecus sp.*) | Human-to-human | [121] |
| | 2003 | Fever, vesiculo-pustular rash | Blood | OPXV-ELISA (IgM, IgG) | ELISA IgG (566/994); ELISA IgM (17/994) | Hunting and carcass preparation | Animal-to-human suspected | [195] |
| | 2007 | Pustular rash, palm and sole lesions | Blood | OPXV-ELISA (IgM. IgG); VZV-ELISA IgG | OPXV-ELISA IgG (3/73); OPXV-ELISA IgM (0/72); VZV-ELISA IgG (7/44); | None reported | None reported | [123] |

| | | | | | | | | |
|---|---|---|---|---|---|---|---|---|
| | | | | | OPXV-ELISA IgG (10/17; smallpox vaccine related) | | | |
| Cameroon | 2017 | No disease symptoms (asymptomatic individuals) | Blood | OPXV-ELISA (IgM, IgG) | OPXV-ELISA IgG (43/125; smallpox vaccinated); OPXV-ELISA IgG (4/63; non-smallpox vaccinated); OPXV-ELISA IgM (1/63) | One person with OPXV-anti IgM titre was involved in selling, cooking, and eating of Gambian rats (*Cricetomys spp.*) and sun squirrels | Animal-to-human transmission suspected in villagers and sanctuary staff | [134] |
| | 2018 | Fever, malaise, body aches, maculopustular rash, pharyngitis or sub-mandibular or cervical lymphadenopathy | Biopsy of skin lesions, swab of skin lesions, blood | OPXV- real-time PCR, Mpox- real-time PCR | OPXV- and Mpox- real-time PCR (1/1; using biopsy, and swab of skin lesions, and blood); OPXV- and Mpox- real-time PCR (0/6; using blood collected between 14-21 days after onset of rash) | None reported | None reported | [108] |
| Sierra Leone | 2007 | None reported | Blood | OPXV-ELISA IgG, OPXV-ELISA IgM | OPXV-ELISA IgG (160/1536); OPXV-ELISA IgG (11/866; non-smallpox vaccinated group); OPXV-ELISA IgM (6/313) | None reported | None reported | [133] |
| | 2017 | Fever, malaise, body aches, vesicular skin | Vesicular swab, blood | Mpox- ELISA IgG, Mpox- real- | Mpox-ELISA IgG (1/1; using | The patient reported hunting | Squirrel-to-human | [109] |

| | | | | | | | | |
|---|---|---|---|---|---|---|---|---|
| | | eruptions, enlarged cervical lymph nodes, dysphagia | | time PCR, Sanger sequencing | day 12 and day 55 blood samples); Mpox- real-time PCR (1/1; using day 12 blood sample); Sanger sequencing (1/1; partial genome) | and feeding upon squirrels ≈ 10 days before the onset of disease | | |
| Sudan | 2005-2006 | Fever, papular rash, vesiculopustular rash, cough, throat and joint pain, lymphadenopathy, skin eruptions, inflammation of nasal mucous membranes, enlarged cervical lymph nodes | EDTA-whole blood, dried blood, vesicular swabs, crust specimens | OPXV- PCR, Mpox- PCR, OPXV-IgG ELISA, OPXV-IgM ELISA, virus isolation, Sanger sequencing | OPXV- and Mpox- PCR (3/19; using samples collected between 5 to 13 days after onset of rash); virus isolation (3/3); OPXV- IgG and IgM ELISA (3/19; samples collected between 25 and 70 days after onset of rash); OPXV- IgG ELISA (4/19; samples collected between 36 and 78 days after onset of rash) | Some of the case patients were suspected of being bitten by an infected animal. No contacts with wild animals were reported by the patients | Human-to-human | [13] |
| USA | 2003 | Rash, fever, chills, cough, sore throat, swollen neck, headache, vomiting, vesicular lesions on palms and soles of feet, lymphadenopathy | Blood | Blood biochemistry, chest, and neck radiographs (X-rays), CT scan, and physical examination | Physical examination and differential diagnosis along with the evidence of Mpox infection in three pet | Child became Mpox positive due to contact with pet prairie dog in the household, the first human case | Prairie dog-to-human | [66] |

| | | | | | | | | |
|---|---|---|---|---|---|---|---|---|
| | | | | | prairie dogs concluded that the child was Mpox positive | of Mpox in non-endemic region | | |
| | 2003 | Fever, malaise, headache, sore throat, dyspnea, vesiculopustular rash, enlarged tonsils, anorexia, malaise, cervical lymphadenopathy, brain edema, encephalopathy, and seizures | Skin lesions, blood, cerebrospinal fluid | OPXV- real-time PCR, Mpox-PCR, OPXV-IgM and IgG ELISA, virus isolation, IHC | OPXV- and Mpox- PCR (3/3; using skin lesions); Virus isolation (3/3; using skin lesions); IHC (2/2; using skin lesions); OPXV-IgM and IgG ELISA (3/3; using sera samples); OPXV-IgM ELISA (1/1; using cerebrospinal fluid); PCR and virus isolation (0/1; using cerebrospinal fluid) | Extensive contacts with two prairie dogs in household. The prairie dogs soon died from illness. | Prairie dog-to-human | [44] |
| | 2003 | Rash, fever, chills, sore throat, cough, sweats, headache, joint pain, keratitis, lymphadenopathy, miscarriage at 12 weeks of gestation | Blood, tissues | PCR, IHC, virus isolation, EM, OPXV- IgM and IgG ELISA | PCR (15/19); Virus isolation (9/19); IHC (12/19); EM (4/19) OPXV-IgM (15/17; using sera collected within 56 days after onset of rash, illness); | Veterinary staff, pet store employees, distributors, and household members, exposed to prairie dogs | Prairie dog-to-human | [11] |

| | | | | | | | | |
|---|---|---|---|---|---|---|---|---|
| | | | | | OPXV-IgG ELISA (15/17) | | | |
| | 2003 | Rash | Skin lesions, acute- and convalescent-phase sera | IgM ELISA, IgG ELISA, PCR, virus isolation | IgM ELISA (34/36; using sera); IgG ELISA (29/36; using sera); PCR (36/36; using lesions); Virus isolation (36/36; using lesions) | None reported | None reported | [124] |
| | 2003 | Fever, chills, sweats, chills, skin lesions, persistent cough, and lymphadenopathy. Cellulitis, pharyngitis, myalgia, diarrhoea, nasal congestion, back pain | Tissues and skin biopsies | PCR, virus isolation, EM, IHC, Sanger sequencing | PCR (6/11); virus isolation (6/11); EM (4/11); IHC (4/11); Sanger sequencing (1/1; complete HA gene) | Distributors and household members, in contact with prairie dogs | Prairie dog-to-human | [65] |
| | 2003 | Rash, fever, lesions, headache, nonpruritic vesicles, lymphadenopathy, encephalitis | Acute- and convalescent-phase sera | IgM ELISA, IgG ELISA | IgM ELISA (1/57) IgG ELISA (3/26) | Contact with prairie dogs | Prairie dog-to-human | [5] |
| | 2003 | Fever, vesicular-pustular rash, sore throat, chills, myalgia, headache, encephalitis, adenopathy | Blood | IgG and IgM ELISA, PCR, IHC, virus isolation | PCR (3/4); IgG ELISA (5/6); IgM ELISA (5/7); IHC (2/2); virus isolation (3/3) | Frequent contact with prairie dogs | Prairie dogs-to-human | [92] |
| | 2004 | None reported | Cutaneous biopsies (retrospective study) | PCR, RFLP, IHC, EM, Sanger sequencing | PCR (3/3; for DNA polymerase gene, and HA gene); EM (3/3; | None reported | None reported | [90] |

| | | | | | | | | |
|---|---|---|---|---|---|---|---|---|
| | | | | | poxvirus particles); IHC (3/3), Sanger sequencing (3/3; complete HA gene) | | | |
| | 2021 | Skin lesions, pustular rash, fever, cough, fatigue, vomiting, diarrhoea | Lesion swab | OPXV- real-time PCR, Mpox-PCR | OPXV- real-time PCR (1/1); Mpox- PCR (1/1) | None reported | Imported case via air travel from Nigeria | [69] |
| | 2021 | Discrete vesicles on forehead and nose, arms, trunk and thighs, cervical lymphadenopathy | Biopsy of an intact pustule, skin lesions | OPXV- real-time PCR, histopathology, virus isolation | OPXV- real-time PCR (1/1); virus isolation (1/1) | No contact with animals | Imported case via air travel from Nigeria | [10] |
| | 2022 | Fever, chills, headache, sore throat, malaise, rash, and pain in rectum | Lesion fluid | Mpox- PCR, IHC | Mpox- PCR (1/1) IHC (0/1; for Herpes simplex virus type 1, Herpes simplex virus type 2, and *Treponema pallidum*) | No contact with animals | None reported | [97] |
| | 2022 | Vesicular lesion, anogenital rash, oral lesion, perianal rash | Lesion swab (vesicular lesion) | OPXV- real-time PCR, Mpox-PCR, NGS | OPXV- real-time PCR (17/17); Mpox- PCR (17/17); NGS (Mpox genome was sequenced for confirmation) | No contact with animals | Human-to-human, attributed to sexual contact | [31] |
| UK | 2018 | Fever, skin rash, lymphadenopathy, maculopustular rash | Skin lesion swabs | Mpox- PCR, Genomic sequencing | Mpox- PCR (2/2); Genomic sequencing (2/2; Mpox vonfirmed) | No contact with animals | Two imported cases from Nigeria. Human-to-human transmission | [71] |

| | | | | | | | | |
|---|---|---|---|---|---|---|---|---|
| | 2018 | Fever, lymphadenopathy, maculopustular rash, headache, sore throat, skin lesions, earache, eye pain | Skin lesion swabs | Mpox- PCR | Mpox- PCR (3/3) | No contact with animals | Human-to-human transmission due to contaminated hospital bedding | [181] |
| | 2018-2021 | Fever, headache, pleiomorphic skin lesions, pustular rash, night sweats, lymphadenopathy, groin swelling, conjunctivitis, pruritis | Blood, nose or throat swab, urine, lesion swab | PCR,<br>OPXV-IFA (IgG and IgM) | PCR (7/7; nose or throat swabs);<br>PCR (6/7; using blood);<br>PCR (4/7; using urine)<br>OPXV-IFA for IgM and IgG (0/4; using blood) | No contact with animals | Index, secondary or tertiary Mpox infections, either locally acquired or exported from Nigeria | [42] |
| | 2019 | Pustular rash, skin lesions | Blood, urine, throat swab and lesion swabs of the patient. Forty-two environmental samples (10 vacuum samples and 32 surface swabs), from patient's and sibling's residences | OPXV- real-time PCR, Mpox- real-time PCR, virus isolation, EM, Illumina MiSeq | OPXV- real-time PCR (1/1);<br>Mpox- real-time PCR (1/1);<br>Mpox- real-time PCR (37/42; using environmental samples);<br>virus isolation (4/4; using different environmental samples);<br>Illumina MiSeq (3/3; complete Mpox genomes, generated using three different samples);<br>EM (1/1; using virus cultured from a towel) | No contact with animals | Imported Mpox case from Nigeria, via air travel. Mpox DNA was detected on multiple surfaces and objects at the patient's and sibling's residences | [105] |

| | | | | | | | | |
|---|---|---|---|---|---|---|---|---|
| | 2021 | Rash, skin lesions | Lesion swabs | PCR, Sanger sequencing | PCR (3/3) Sanger sequencing (3/3; confirmed Mpox) | No contact with animals | Human-to-human transmission of imported Mpox from Nigeria, in a family | [7] |
| Israel | 2018 | Maculopapular rash on face, trunk, neck, lower and upper extremities, lesions on soles and palms, chills, two ulcers on penis shaft, bilateral enlarged and tender lymph nodes in groin, one lesion on posterior aspect of left arm, fever | Scab, pustule, blood | PCR, EM, OPXV-IFA IgG, virus isolation, high throughput sequencing | PCR (1/1); EM (1/1); OPXV-IFA IgG (1/1); virus isolation (1/1); genome sequencing (1/1) | Patient discarded two rodent carcasses few days before the onset of disease symptoms | Patient had a travel history to Nigeria. Suspected transmission from rodent carcasses to human | [110] |
| | 2018 | Fever, skin lesions | Pustule swabs | PCR, Nanopore MinION and Illumina MiSeq sequencing | PCR (1/1); Nanopore MinION and Illumina MiSeq (1/1; generated complete genome) | Exposed to carcasses of dead rodents in Nigeria before travelling | Suspected transmission from rodent carcasses | [4] |
| | 2022 | Skin lesions | Oropharyngeal swabs, skin lesion exudate swabs, rectal swabs | Mpox- real-time PCR, plaque assay | Mpox- real-time PCR (32/32). Skin lesion swabs generated lower Cq values or higher viral load than oropharyngeal swabs for most patients | None reported | None reported | [145] |
| Italy | 2022 | Skin and genital lesions, inguinal lymphadenopathy, | Skin, genital and anal lesions, serum, plasma, | OPXV- real-time PCR, Mpox- PCR, Sanger | OPXV- real-time PCR (4/4); Mpox- PCR (4/4); | No contact with animals | Human-to-human, | [116] |

| | | | | | | | | |
|---|---|---|---|---|---|---|---|---|
| | | fever, asthenia, myalgia | seminal fluid, feces, nasopharyngeal swabs | sequencing, metagenomic NGS using ION Torrent PGM; Sanger sequencing | ION Torrent PGM (1/1; complete Mpox genome); Sanger sequencing (1/1; complete HA gene) | | attributed to sexual contact | |
| | 2022 | Fever, skin lesions, maculopapular lesions | Skin lesion, plasma, serum, semen, urine | Mpox- real-time PCR, virus isolation, IFA | Mpox- real-time PCR (1/1; using skin lesion sample); IFA (1/1; detected IgG and IgM antibodies in serum on day 8 after onset of symptoms); Virus isolation (1/1; using semen) | No contact with animals. The study noted that Mpox DNA was detected in semen samples for longer duration than in skin lesions. | Human-to-human, attributed to sexual contact | [138] |
| | 2022 | Fever, skin lesions, oropharyngeal lesions | Oropharyngeal and skin lesion swabs, anal swab, urethal swab | OPXV- real-time PCR, Mpox- real-time PCR, virus isolation | OPXV- real-time PCR (33/33); Mpox- real-time PCR (33/33); virus isolation (using anal and uretral swabs) | While most patients were symptomatic, two were asymptomatic with no rash. The presence of Mpox DNA in urethral and anal swabs suggested transmission via sexual contact. | Human-to-human, attributed to sexual contact | [95] |
| | 2022 | Fever, sore throat, headache, fatigue, inguinal lymphadenomegaly, vesicular rash, skin lesions, | Pustule swab, nasopharyngeal swab | Mpox- real-time PCR | Mpox- real-time PCR (1/1) | No contact with animals. A patient was coinfected with Mpox, SARS-CoV-2, and HIV. | Human-to-human, attributed to sexual contact | [175] |

| | | | | | | | | |
|---|---|---|---|---|---|---|---|---|
| | | pharyngodynia, pustules | | | | | | |
| | 2022 | Pustular lesions, asthenia, headache, myalgia, chills, inguinal lymphadenopathy | Blood | Mpox- real-time PCR | Mpox- real-time PCR (1/1) | No contact with animals | Human-to-human, attributed to sexual contact | [177] |
| Spain | 2022 | Fever, skin lesions, influenza-like illness, sore throat, headache, lymphadenopathy (cervical, inguinal, axillary), proctitis, tonsillitis, exanthem | Throat swabs, skin swabs, anal swabs | Mpox- real-time PCR | 181 Mpox- real-time PCR positive cases; Mpox- real-time PCR (178/180; 99% cases were PCR positive using oral swabs, 82/117; 70% cases were PCR positive using throat swabs, and 43/55; 78% cases were PCR positive using anal swabs | 181 PCR confirmed cases of Mpox in Spain were investigated for transmission dynamics. 166/181 of positive cases were amongst patients who identified as MSM. Skin lesions were the most common clinical disease symptom in these cases. A large number of cases also had proctitis | Human-to-human, attributed to sexual contact | [176] |
| | 2022 | Skin lesions, myalgia, fatigue, odynophagia, malaise, proctitis, proctalgia, headache | Saliva, skin lesion swabs, nasopharyngeal swabs, rectal swabs, urine, semen, feces | OPXV- real-time PCR, Mpox- real-time PCR, Sanger sequencing | Mpox-real-time PCR (12/12); Mpox- real-time positive: saliva (12/12); skin lesions (12/12); rectal swabs (11/12); nasopharyngeal swabs (10/12); urine (9/12); feces | No contact with animals | Human-to-human, attributed to sexual contact | [178] |

| | | | | | | | | |
|---|---|---|---|---|---|---|---|---|
| | | | | | (8/12); semen (7/9); Sanger sequencing (3/3; partial genomes) | | | |
| | 2022 | Fever, asthenia, myalgia, inguinal lymphadenopathy, proctitis, urethritis, rash, nasal congestion, cough | Vesicular lesion swabs | OPXV- real-time PCR, Mpox- PCR, EM, Illumina sequencing | OPXV- real-time PCR (48/48); Mpox- real-time PCR (48/48); Illumina sequencing (14/14; complete Mpox genomes); EM (1/1; poxvirus particles) | Samples obtained during first nine days after onset of clinical symptoms had highest viral loads with Ct < 25. Total 14 complete genomes from samples with Ct < 30 were sequenced. | Most Mpox confirmed cases (87.5%) were attributed to human-to-human transmission via sexual contact | [96] |
| | 2022 | Fever, rash exanthema, lymphadenopathy, asthenia, myalgia, headache, odynophagia, proctitis | Vesicular lesions, urine, pharyngeal exudates | Mpox- real-time PCR, Sanger sequencing | Mpox- real-time PCR (595/802); Sanger sequencing (2/2; partial genome) | Most cases included MSM. Several cases also had HIV coinfection | Human-to-human, attributed to sexual contact | [86] |
| | 2022 | Fever, proctitis, headache, myalgia, arthralgia, lymphadenopathy, pustular rash, maculopapular rash, perianal ulcers | Pustule swab | Mpox- real-time PCR | Mpox- real-time PCR (1/1) | The patient had existing HIV infection | Human-to-human, attributed to sexual contact | [174] |
| | 2022 | Fever, rash, lymphadenopathy, asthenia, sore throat, myalgia, vomiting, diarrhoea | Skin lesions | OPXV- real-time PCR | OPXV- real-time PCR (16/16; pediatric patients) | No contact with animals. Contact with contaminated material, household contact | Human-to-human transmission | [172] |

| | | | | | | | | |
|---|---|---|---|---|---|---|---|---|
| Portugal | 2022 | Fever, lesions on genitals, rash, genital ulcers, exanthema, inguinal lymphadenopathy | Exudates or swabs from lesions, crusts | OPXV- PCR, Sanger sequencing, Nanopore MinION sequencing | OPXV- PCR (96/145); Sanger sequencing (partial Mpox genomes); Nanopore MinION sequencing (1/1) | No contact with animals | Human-to-human, attributed to sexual contact | [117] |
| | 2022 | Fever, macular rash, inguinal lymphadenopathy, skin lesions | Lesion swab | Mpox- PCR | Mpox- PCR (1/1) | No contact with animals | Human-to-human, attributed to sexual contact | [94] |
| France | 2022 | Fever, intense fatigue, chills, myalgia, severe anal pain, sore throat, enlarged cervical lymph nodes | Pharyngeal swab | OPXV- real-time PCR | OPXV- real-time PCR (1/1) | No contact with animals | Human-to-human, attributed to sexual contact | [179] |
| Belgium | 2022 | Vesicular perianal rash (1 patient only); other 3 patients were asymptomatic while being HIV positive | Oropharyngeal swabs, anorectal swabs, urine, convalescent-phase sera | OPXV- PCR, Mpox- PCR, virus isolation, Nanopore MinION sequencing, IFA | OPXV- PCR (4/224); Mpox- PCR (4/224); Virus isolation (2/3); MinION sequencing (1/1); IFA IgG (2/2). Anorectal swabs were PCR positive on day 0 but negative for Mpox DNA on days 21, 24, and 37 | A retrospective analysis of patient samples visiting a sexual clinic. The patients were suspected of having suffered from gonorrhoea/ chlamydia infections. One patient with painful vesicular perianal rash was misdiagnosed as a flare-up of herpes simplex | Human-to-human, attributed to sexual contact | [98] |
| Germany | 2022 | Fever, malaise, fatigue, myalgia, sweats, | Fluid from cutaneous | PCR | PCR (6/6; cutaneous blisters); | No contact with animals. Six | Human-to-human, | [146] |

| | | | | | | | | |
|---|---|---|---|---|---|---|---|---|
| | | lymphadenopathy, anal pain (occurred due to anal ulcer, anal abscess, anal fissure, proctitis, and retal ulcer) | blisters, seminal fluid | | PCR (1/1; seminal fluid) | cases among MSM | attributed to sexual contact | |
| | 2022 | Pustular rash | Skin lesion | Mpox- PCR | Mpox- PCR (1/1) | No contact with animals. The patient was coinfected with HIV and syphilis | None reported | [180] |
| | 2022 | Fever, malaise, joint, muscle and back pain, headache, papular skin lesions, fatigue, cough, inguinal lymphadenopathy, anal pain, vesicular skin lesions | Pustule swab, serum, oral lesion swab, plasma, semen, urine | OPXV- real-time PCR, Mpox- real-time PCR, virus isolation, Illumina sequencing, IFA | OPXV- real-time PCR (2/2); Mpox- real-time PCR (2/2); All samples except urine had detectable Mpox DNA. Virus isolation (1/1; using pustule swab, blood, and semen); IFA IgG (detectable only after 2 weeks of hospital admission) | No contact with animals. One patient was co-infected with HIV | Human-to-human, attributed to sexual contact | [99] |
| Switzerland | 2022 | Cutaneous lesions on a lip and chin, conjunctivitis, and itching eye (unidirectional) | Swabs of cutaneous lesions on the chin and one lip, conjunctivital swab | Mpox- PCR | Mpox- PCR (1/1; using swabs of cutaneous lesions and conjunctivitis) | The conjunctivital swab was Mpox positive. It was suspected that the patient acquired Mpox infection during opthalmologic visit to the eye clinic | Suspected infection during opthalmologic visit to the eye specialist | [100] |

| | | | | | | | | |
|---|---|---|---|---|---|---|---|---|
| Greece | 2022 | Fever, rash, fatigue, myalgia | Swab of the rash | Mpox- PCR | Mpox- PCR (1/1) | No contact with animals. The patient had recently visited Portugal | Human-to-human, via sexual activity | [148] |
| India | 2022 | Fever, myalgia, maculopapular rash, skin lesions, enlarged cervical and inguinal lymph nodes, backache, headache, chills, cervical lymphadenopathy | Oropharyngeal swabs, nasopharyngeal swabs, blood, urine, lesion fluid, skin lesions | OPXV- real-time PCR, Mpox- real-time PCR, Illumina sequencing | OPXV- real-time PCR (2/2); Mpox- real-time PCR (2/2). All clinical samples of both patients except blood of patient 2 were MPXV positive. Illumina sequencing (2/2; cgenerated omplete genomes) | Two travelers from UAE to India were Mpox positive | Human-to-human | [104] |
| South Korea | 2022 | Fever, chills, sore throat, headache, skin lesions, perioral erosive lesions, maculopustular rash, penile ulcer | Skin lesion, oropharyngeal swab, nasopharyngeal swab, blood, crust specimen | Mpox- real-time PCR, conventional PCR, Sanger sequencing | Mpox- real-time PCR (1/1; using skin lesions and swab samples); Conventional PCR (1/1; using skin lesions and swab samples); Sanger sequencing (1/1) | First Mpox case of South Korea. A traveller (Korean national) who arrived in South Korea from Germany | None reported | [93] |
| | 2022 | Skin lesions | Skin lesion, oropharyngeal swab, nasopharyngeal swab | MPXV- PCR, OPXV- PCR, virus isolation, EM, Sanger sequencing | MPXV- PCR (1/1); OPXV- PCR (1/1); virus isolation (1/1; using skin lesion, oropharyngeal and | No contact with animals. First isolation of Mpox in South Korea | None reported | [140] |

| | | | | | | | | |
|---|---|---|---|---|---|---|---|---|
| | | | | | nasopharyngeal swabs); EM (1/1; poxvirus particles); Sanger sequencing (1/1; generated partial genome) | | | |
| Australia | 2022 | Genital rash and lesions, skin lesions, pustules, fever, malaise | Rectal swab, throat swab, urine, swabs of deroofed skin lesions from the hand, calf, and trunk, combined nose-throat swabs | VZV- real-time PCR, HSV- real-time PCR, Mpox-PCR, OPXV- real-time PCR, Mpox- real-time PCR, virus isolation, EM, Illumina sequencing | VZV- real-time PCR (0/1; using urine, rectal and throat swab); HSV- real-time PCR (0/1; using urine, rectal and throat swab); OPXV- real-time PCR (1/1; using skin lesions and nose-throat swab); Mpox- real-time PCR (1/1; using skin lesions and nose-throat swab); Virus isolation (1/1; using lesion swabs on hand and abdomen); EM (1/1; poxvirus particles); Illumina sequencing (1/1; generated whole genome) | Initial diagnosis was gonorrhea, then suspected superimposed bacterial cellulitis. Coinfection with HIV | Human-to-human, via sexual activity | [14] |

| | | | | | | | | |
|---|---|---|---|---|---|---|---|---|
| Multi-country outbreaks (16 countries) | 2022 | Rash, skin lesions, fever, pharyngitis, lymphadenopathy, headache, fatigue, myalgia, proctitis or anorectal pain | Throat swabs, skin lesions | Mpox- real-time PCR | Mpox- real-time PCR (528 confirmed cases in 16 countries). A subset of 32 cases: Mpox- real-time PCR (22/32; using skin lesion samples, and 20/32 using throat swab samples) | There were 528 Mpox PCR confirmed cases in 16 countries during April-June 2022. This study reported a subset of 32 cases, 30 of which were cases including MSM | Human-to-human, attributed to sexual contact | [48] |

CAR = Central African Republic; DRC = Democratic Republic of the Congo; EM = Electron microscopy; HI = Hemagglutination inhibition; HSV = Herpes Simplex virus; IFA = Immunofluorescence, IgG = Immunoglobulin G; IgM = Immunoglobulin M; IHC = Immunohistochemistry; MSM = men who have sex with men; OPXV = Orthopoxvirus; PCR = Polymerase chain reaction; UAE = United Arab Emirates; UK = United Kingdom; USA = United States of America; VZV = Varicella zoster virus.
