# Supplementary material for: Overview of Diagnostic Methods, Disease Prevalence and Transmission of Mpox (Formerly Monkeypox) in Humans and Animal Reservoirs
Source: Microorganisms. 2023 Apr 30;11(5):1186. doi: 10.3390/microorganisms11051186 (PMC10223876; doi:10.3390/microorganisms11051186)
Supplement: Supplementary file 1 [file microorganisms-11-01186-s001.zip › Supplementary Table S2.pdf]

Supplementary Table S2: Data on Mpox disease in animals, including the prevalence, samples and methods used for detection, and clinical signs

Country	Duration of study	Mpox positive animal species- Common name (Scientific name)	Clinical signs of disease in animals	Samples used for detection	Summary of results	Notes/ Special observations	Citations
Denmark	1958	Cynomolgus monkey (*Makaka fascicularis*)	Maculopapular rash and skin lesions	Lesion swab	HI test- OPXV antibodies (17/152); virus isolation (3/3); EM (1/1; poxvirus particles)	The first report of Mpox in captive monkeys at the Stetens Serum Institut, Copenhagen	[51]
DRC	1971	Monkey (*Cercopithecus mona wolfi*), chimpanzee (*Pan troglodytes verus*)	None reported	Blood, organs of the monkey and chimpanzee (liver, spleen, and kidneys)	Sera samples obtained from 1/1 monkey (*Cercopithecus mona wolfi*) and 1/1 chimpanzee (*Pan troglodytes verus*) were positive for OPXV antibodies with HI, neutralization, and agar gel microprecipitation assays. Virus isolation for poxvirus was confirmed from kidney of chimpanzee that was positive for OPXV serology	None	[58]
	1971	Langur monkey (*Macaca philippinensis*), cynomolgus monkey (*Macaca fascicularis*), chimpanzee (*Pan troglodytes verus*)	None reported	Organ samples (kidneys) of monkeys and a chimpanzee	6/6 kidney samples taken from langur monkeys (*Macaca philippinensis*), and cynomolgus monkeys (*Macaca fascicularis*) were poxvirus positive with virus isolation and HI assay. 1/1 kidney sample of chimpanzee was also poxvirus positive with virus isolation and HI assay	Kidney samples taken from sick as well as healthy appearing monkeys and a chimpanzee were poxvirus positive using virus isolation and HI assay	[152]
	1985	Squirrel (*Funisciurus anerythrus*)	Skin eruptions	Blood, skin, and organ samples of a dead squirrel and blood from live squirrels	Mpox- antibodies were detected in 3/18 squirrels by RIA. Mpox was isolated from skin, lung, spleen, and kidney samples taken from a dead wild squirrel. While 3 squirrels had Mpox antibodies, one of them had active Mpox infection	This was the first report of Mpox isolation from a squirrel	[151]
	1996-1997	Domestic pig (*Sus scrofa*), Gambian rat (*Cricetomys emini*),	None reported	Blood	OPXV- ELISA detected OPXV neutralizing antibodies in 1/1 domestic pig (*Sus scrofa*), 3/19 Gambian rats (*Cricetomys emini*), 1/3 elephant shrew	None	[113]

		Elephant shrew (*Petrodromus tetradactylus*), Thomas's tree squirrel (*Funisciurus anerythrus*), Kuhl's tree squirrel (*Funisciurus congicus*), Sun squirrel (*Heliosciurus rufobrachium*)			(*Petrodromus tetradactylus*), 2/4 Thomas's tree squirrel (*Funisciurus anerythrus*), 7/18 Kuhl's tree squirrel (*Funisciurus congicus*), and 1/2 sun squirrel (*Heliosciurus rufobrachium*); total 15/59 animals		
	2010-2016	Thomas's rope squirrel (*Funisciurus anerythrus*),<br>Lunda rope squirrel (*Funisciurus bayonii*),<br>Target rat (*Stochomys longicaudatus*),<br>Giant pouched rat (*Cricetomys sp.*),<br>Shrew (*Crocidura littoralis*)	None reported	Kidney, liver, and spleen tissue samples from euthanized animals	OPXV- real-time PCR (5/256);<br>Mpox- PCR (5/5);<br>Illumina NextSeq (Complete and near complete Mpox genomes were generated from *Funisciurus anerythrus*, *Funisciurus bayonii*, and *Crocidura littoralis*).<br>Sanger sequencing (Partial genomes were generated from *Stochomys longicaudatus*, and *Cricetomys sp.*)	Two PCR assays targeting different genes, including P4A for OPXV and hemagglutinin for Mpox, PCR positives were confirmed with Sanger sequencing	[111]
	2012-2015	Rope squirrel (*Funisciurus spp.*), African dormouse (*Graphiurus lorraineus*), giant-pouched rat (*Cricetomys emini*), sun squirrels (*Heliosciurus sp.*), rusty-nosed rats (*Oenomys hypoxanthus*), and elephant shrews (*Petrodromus tetradactylus*)	None reported	Blood, oral swabs, lesions, tissues of liver, lung, spleen, heart, kidney, and brain.	OPXV- PCR (0/353);<br>OPXV-ELISA IgG (7/353): anti-OPXV IgG antibodies were detected in<br>2/6 rope squirrels (*Funisciurus spp.*), 1/13 African dormouses (*Graphiurus lorraineus*), 1/9 giant-pouched rats (*Cricetomys emini*), 1/3 sun squirrels (*Heliosciurus sp.*), 1/22 rusty-nosed rats (*Oenomys hypoxanthus*), and 1/17 elephant shrews (*Petrodromus tetradactylus*)	None	[122]

Ivory Coast, Nigeria	1976	Cynomolgus monkey (*Makaka fascicularis*), African monkey (*Cercopithecus spp*.)	None reported	Blood	4/13 African monkey sera were Mpox positive by neutralization assay and IFA	None	[126]
West Africa	1977	Red colobus monkey (*Colobus badius*), lesser white-nosed monkey (*Cercopithecus petaurista*)	None reported	Blood	Mpox- HI titers were detected in 1/15 Red colobus monkeys (*Colobus badius*) and 2/31 lesser-white-nosed monkeys (*Cercopithecus petaurista*)	Wild caught monkeys were examined for serologic investigation of Mpox	[125]
USA	2003	Prairie dog (*Cynomys sp*.)	Anorexia, ocular and nasal mucous discharge, oral ulcers, skin nodules, depression, respiratory distress	Organ tissues	Real-time PCR (1/1); virus isolation (1/1); EM (1/1; poxvirus particles)	Prairie dog was sick and therefore euthanized for necroscopic observation	[91]
	2003	Prairie dog (*Cynomys sp*.)	Ocular discharge, lymphadenopathy, and papular skin lesions. The prairie dog later died after one week of illness	Submandibular lymph node	Mpox- PCR (1/1); virus isolation (1/1); EM (1/1; poxvirus particles in tissue culture were visible)	Initially a gram-negative bacillus was isolated, raising the suspicion of tularemia or plague. The EM identified poxvirus particles in skin biopsy tissues	[65]
	2003	Prairie dog (*Cynomys sp*.), Gerbil (*Gerbillus sp*.), Chinchilla (*Chinchilla sp*.), Hamster (*Cricetus spp*.),	No disease symptoms reported. Animals were collected from exotic pet dealers and private homes, euthanized for investigation	Organ tissues	Real-time PCR (12/52 animals- 3 prairie dogs, 2 Hamsters, 6 Gerbils, and 1 Chinchilla) were Mpox DNA positive using different organ tissues. Sanger sequencing of amplicons (139 bp for N3R gene and 107 bp for F3L gene) confirmed Mpox infection	Mpox outbreak associated with small mammals imported to the United States in 2003	[89]
	2003	Prairie dog (*Cynomys sp*.)	Of 15 sick prairie dogs, 10 quickly died, while remaining five animals exhibited anorexia, wasting,	Lung and tongue tissues	Novel multiplex real-time PCR (2/2); virus isolation (2/2); Histopathology detected ulcerated lesion of palpebral conjunctiva in eyelid; OPXV lesions were detected using OPXV-IHC assay.	Novel multiplex real-time PCR assay detected OPXV- DNA polymerase gene (E9L, non-variola) and Mpox- envelop gene (B6R), while the standard	[64]

			coughing, sneezing, ocular discharge, and swollen eyelids. Initial diagnosis suspected tularemia and therefore two prairie dogs were euthanized for pathologic investigation		Virus isolation (2/2). EM detected poxvirus particles	singleplex PCR could not detect Mpox. Specific Mpox real-time PCR detected Mpox DNA	
	2003	African dormouse (*Graphiurus spp*.), Giant pouched rat (*Cricetomys spp*.), African hedgehog (*Atelerix spp*.), Jerboa (*Jaculus spp*.), Black-tailed prairie dog (*Cynomys ludovicianus*), Gray short-tailed opossum (*Monodelphis domestica*), Southern opossum (*Didelphis marsupialis*), Rope squirrel (*Funisciuris spp*.), Woodchuck (*Marmota monax*)	No apparent disease symptoms were observed in traded animals from Ghana (the exotic pet trade)	Total 18 sample types, including oral swab, ocular swab, eyelid, tongue, lesion, blood, serum, heart, lung, liver, spleen, kidney, abdominal skin, lymph node, gonad, urine, feces, and brain were collected from each animal	Mpox-specific real-time PCR positive animals: African dormouse: 9/37, Giant pouched rat: 2/12, African hedgehog: 1/21, Jerboa: 1/4, Black-tailed prairie dog: 14/18, Gray short-tailed opossum: 1/4, Southern opossum: 1/6,<br>Rope squirrel: 3/11, Woodchuck: 1/4. (Total 33 animals were Mpox positive for Mpox DNA).<br><br>OPXV- ELISA IgG positive animals: African dormouse: 2/25, Giant pouched rat: 5/11, African hedgehog: 2/45, Southern opossum: 1/3.<br><br>Mpox tissue culture positive animals: African dormouse: 8/9, Giant pouched rat: 1/10, Black-tailed prairie dog: 9/14, Southern opossum: 1/1, Rope squirrel: 3/6. Total 22/30 animals that were PCR positive were also positive for tissue culture.	The eyelid (10/10), skin (10/10), and lymph nodes (10/10) were uniformly positive for Mpox DNA for 10/14 prairie dogs that were Mpox positive.<br><br>Blood (6/6), eyelid (8/8), gonad (8/8), heart (9/9), kidney (9/9), liver (9/9), lung (8/8), lymph node (4/4), skin (9/9), and urine (4/4) samples were Mpox DNA positive for dormice.	[153]
Ghana	2004	African dormice (*Graphiurus spp*.), Giant pouched rat (*Cricetomys sp*.), African ground squirrel (*Xerus sp*.)	None reported	Organ tissues	OPXV- real-time PCR:<br>6/97 *Graphiurus spp*., 2/38 *Cricetomys sp*., 1/1 *Xerus sp*.<br>OPXV- ELISA IgG:<br>9/90 *Graphiurus sp*., 1/38 *Cricetomys sp*., 2/5 *Funisciurus sp*., and 1/7	Virus isolation from animal tissue samples that were OPXV positive by real-time PCR were negative in cell culture	[120]

					Heliosciurus sp. had variable levels of OPXV antibodies		
Uganda	2006	Red colobus monkey (*Colobus badius*)	None reported	Blood	OPXV- ELISA IgG (8/31; using vaccinia virus antibodies); Postadsorption ELISA to differentiate among vaccinia, Mpox, and cowpox virus antibodies was inconclusive and had equal cross reactivity. Western blot analysis was more informative than ELISA	Plaque reduction assay suggested that all the 31 monkeys may have become infected with Mpox-like poxvirus, but the serology could not confirm the virus	[149]
Zambia	2009-2013	Vervet monkey (*Chlorocebus pygerythrus*), yellow baboon (*Papio cynocephalus*), chacma baboon (*Papio ursinus*), shrew (*Crocidura sp*.), rodent (*Mastomys sp*.), rodent (*Steatomys sp*.), rodent (*Cricetomys sp*.)	None reported	Blood and organ tissues of monkeys, spleen tissues of rodents and shrews	OPXV- real-time PCR:<br>0/88 vervet monkeys (*Chlorocebus pygerythrus*); 0/50 yellow baboons (*Papio cynocephalus*); 0/50 chacma baboons (*Papio ursinus*); 0/259 rodents; 0/42 shrews.<br><br>OPXV IgG ELISA:<br>4/50 chacma baboons (*Papio ursinus*); 14/42 shrews (*Crocidura sp*.); 33/173 rodents (*Mastomys sp*.); 4/15 rodents (*Steatomys sp*.); and 1/15 rodents (*Cricetomys sp*.)	None	[150]
Ivory Coast	2016-2017	Sooty Mangabey (*Cercocebus atys*)	Skin lesions in a dead Sooty Mangabey	Blood, skin lesion, tissues from spleen, lung, kidney, skin, liver, heart, thymus, intestine, muscles, throat swabs, and lymph nodes	OPXV- real time PCR (The Sooty Mangabey (*Cercocebus atys*) was OPXV positive in all tissues, skin lesions, and throat swab except for muscle tissues).<br><br>IFA IgG and IgM (1/1);<br>virus isolation followed by genome sequencing using ION Torrent PGM was successful for all samples	A dead Sooty Mangabey was found; skin Lesions noted. The OPXV DNA was detected in various tissue samples, skin lesions, and throat swab except for the muscle tissues	[106]
	2016-2017	Nonhuman primates- Chimpanzee (*Pan troglodytes verus*)<br><br>Insects- maggots and	Respiratory illness exhibiting coughing, breathing through open mouth, nasal,	Nonhuman primates (NHP)- feces, urine. Fruit-wedges, flies,	62/492 NHP feces were Mpox positive by PCR; 11/55 urine samples were Mpox positive by PCR; 5/26 fruit-wedges were Mpox positive by PCR; 4/80 flies were Mpox positive by PCR;	Mpox-positive samples with high virus copy number were used for virus isolation and genome sequencing.	[107]

		Flies, feeding upon a chimpanzee corpse	and ocular discharge, a single vesicular lesion, rash	fly feces, maggots from a NHP corpse, tissues from NHPs (liver, lung, spleen, skin, stomach, jejunum, colon, bladder, testis)	1 maggot was Mpox positive by PCR; 9 necropsy samples were Mpox positive by PCR. Virus isolation was attempted for those samples that had high virus copy numbers. Only 2/12 samples were successful for virus isolation; one from a leaf swab and other from NHP feces. Illumina MiSeq and Sanger sequencing generated 17/17 partial or complete Mpox genomes	Mpox genomes were generated from 14 NHP feces, 1 leaf swab, 1 fruit-wedge, and 1 virus isolate	
Barbados	2019	Wild mice (*Mus musculus*), Wild rat (*Rattus norvegicus*, *Rattus rattus*)	None reported	Blood	OPXV- IFA IgG: *Rattus rattus* (1/2); *Rattus norvegicus* (1/8); *Mus musculus* (10/150)	None	[130]
France	2022	Dog (*Canis lupus familiaris*)- Greyhound; 4-years-old, male	Mucocutaneous lesions, abdomen pustules, anal ulcer	Skin lesions, anal swab, oral cavity swab	Mpox- specific real-time PCR (1/1; using skin lesion, anal, and oral cavity swab specimens); Nanopore MinION sequencing (1/1; generated Mpox genome from the dog)	The Mpox genome generated from dog was 100% identical with another genome that was generated from a human in the household, suggested human to dog transmission of Mpox	[154]

ELISA = Enzyme linked immunosorbent assay; EM = Electron microscopy; HI = Hemagglutination inhibition; IFA = Immunofluorescence assay; IgG = Immunoglobulin G; IgM = Immunoglobulin M; IHC = Immunohistochemistry; NHP = Nonhuman primate; OPXV = Orthopoxvirus; PCR = Polymerase chain reaction; RIA = Radioimmunoassay; USA = United States of America
